# Report of Diseases in the Public and Private Practice of One of the Physicians of the Finsbury Dispensary, from the 20th of August to the 20th of September

**Published:** 1805-10-01

**Authors:** J. Reid

**Affiliations:** Grenville Street, Brunswick Square


### *Report of Diseases in the public and private Practice of one of the Physicians of the Finsbury Dispensary, from the 20th of August to the 20th of September.*

| | | | |
|---|---|---|---|
| Apoplexia - - - - - | 1 | Cynanche - - - - - - | 9 |
| Dyspepsia - - - - | 11 | Morbi infantiles - - - | 22 |
| Hypochondriasis - - - | 9 | Morbi cutanei - - - | 8 |
| Anasarca - - - - - - | 5 | Diarrhœa et Cholera - | 13 |
| Hydrothorax - - - - | 3 | Menorrhagia - - - - - | 5 |
| Dyspnœa ebriosa - - | 1 | Amenorrhœa et Chlorosis | 12 |
| Phthisis pulmonalis - - | 10 | Epilepsia - - - - - - | 1 |
| Catarrhus - - - - - | 15 | Asthenia - - - - - - | 16 |

A few

A few days since the Reporter was called to a patient that had been seized with an attack of apoplexy. Unfortunately, before his arrival, the patient had been bled. The disease was occasioned by an extraordinary degree of bodily exertion, which was followed almost immediately by an excessive and unseasonable exercise of the mind. From the cause that produced it, independently of the symptoms that it exhibited, the state of the person afflicted was evidently that of extreme debility and exhaustion.

There are few instances, one should imagine, in which a person, whose understanding has not been debauched by superannuated prejudice, or practice been enslaved by the trammels of a professional and hereditary routine, would think of removing debility by abstracting blood, or of restoring an enfeebled and exhausted frame, by evacuating any part of that fluid which conduces most essentially and immediately to its vigour and support.

The fatal result of apoplexy, perhaps, too frequently arises from the manner in which it is treated.* Sometimes, even after a paroxysm has subsided, bleeding is had recourse to, from a vague and empirical notion of its indiscriminate utility in this disease.

Let it not, however, be misunderstood as the Reporter's opinion, that there are not many cases of this disease which do, but merely that there are many which do *not* require and admit the remedy of venæsaction; a remedy, the immediate application of which is *often* essential to the salvation of the patient.

The former cases are, for the most part, characterized by a high degree of excitement, arising from the operation of violent stimuli, physical or mental, before their second effect of indirect debility has had time to take place;

* An example from Dr. Whytt might have been introduced in the text, as illustrating the danger attendant upon blood-letting, in every case of real or imaginary apoplexy.

"A delicate or nervous girl having chilled herself at the return of a critical period, was next morning, at four o'clock, seized with stupor, and difficulty of speaking or moving. She was soon after blooded and blistered. At eight o'clock she could neither speak nor swallow, had a hiccup, and was pale and cold, though her pulse and breathing were natural. About half after ten she began to breathe hard, and with a snorting noise. Besides taking medicines, she was now blooded again, and a third time in the afternoon, and died at ten o'clock, eighteen hours after her first seizure." This is a fair instance of mere nervous debility, and deficient excitement, being converted, by means employed for its removal, into a case of genuine and fatal apoplexy.

place; such as what originates from any agony or extacy, more especially from an impetus of anger, which, in a constitution predisposed, is more apt than any other to precipitate an attack of apoplexy.

A person, therefore, inclined to this disease should be particularly assiduous in studying the science of self-government; and those who are connected with him, ought to be anxiously afraid of giving rise to any unnecessary cause of fretfulness or irritation.†

The mode of dress is not sufficiently attended to by persons liable to the complaint of which we have been treating. All tight ligatures, more especially any about the neck, should be carefully avoided. Dress, in the prevention of disease in general, or in relief of morbid habits already established, has not, perhaps, been sufficiently attended to. Remarks, with regard to this subject, may now appear less important and appropriate, as the straight and distorting habiliments of the male, and more especially of the female sex, have apparently been laid aside. But, in the latter, "the old plan of severe constriction, much oftener than is expected, lurks below the free Grecian flow of the external habit." And it ought likewise to be remarked, that the recent passion for almost semi-nakedness, in this age of exquisite polish and refinement, is much more inconsistent with health, and scarcely less so with delicacy and decorum, than that nearly entire exposure which, according to the report of history, characterized the original and indigenous barbarians of our island.

J. REID.

*Grenville Street, Brunswick Square, Sept. 24, 1805.*

† A pampered and podagric Nabob, in one of the modern comedies, upon some provoking opposition, exclaims, "the Doctors order I should never be contradicted!" Ludicrous as this peevish exclamation may appear in the play, such advice might be seriously and judiciously given to the friends or attendants of a gouty, or what is nearly akin, an apoplectic patient.

MEDICAL

